# Circulating cytokines as triggers of endothelial dysfunction and sex-specific interstitial cell response in fibrocalcific aortic valve disease

**DOI:** 10.7150/ijbs.125469

**Published:** 2026-04-23

**Authors:** Vincenza Valerio, Francesca Bertolini, Valentina Rusconi, Mattia Chiesa, Ilaria Massaiu, Paola Gripari, Valentina Mantegazza, Cristina Gatto, Michele Ciccarelli, Arianna Galotta, Alice Bonomi, Marco Zanobini, Marco Agrifoglio, Veronika A. Myasoedova, Paolo Poggio

**Affiliations:** 1 Centro Cardiologico Monzino IRCCS, 20138 Milan, Italy.; 2 University of Naples “Federico II”, Department of Pharmacy, 80138 Naples, Italy.; 3 University of Salerno "Scuola Medica Salernitana", Department of Medicine, Surgery and Dentistry, 84081 Baronissi, Italy.; 4 University of Salerno "Scuola Medica Salernitana", Scuola di Specializzazione in Patologia Clinica e Biochimica Clinica, 84081 Baronissi, Italy.; 5 University of Milan, Department of Biomedical, Surgical, and Dental Sciences, 20122 Milan, Italy.

## Abstract

**Background:**

Fibrocalcific aortic valve disease (FCAVD) is a progressive and multifactorial pathology that remains asymptomatic in its early stages and lacks effective pharmacological therapies. Aortic valve sclerosis (AVSc), the initial phase of FCAVD, is marked by leaflet thickening and early calcium deposition without significant hemodynamic changes. While local inflammation is known to drive valvular remodeling, recent studies suggest that systemic inflammation may also play a critical role, potentially interacting with endothelial (VEC) and interstitial cells (VIC) and thus promoting disease progression. Notably, sex differences in fibrocalcific processes have been identified, yet their mechanistic basis remains understudied. Thus, we hypothesize that systemic inflammation exacerbates endothelial dysfunction and accelerates fibrocalcific remodeling, with distinct processes in men and women, and aim to investigate how these mechanisms contribute to disease progression.

**Methods:**

A total of 238 individuals were enrolled across three groups: controls (CTRL n = 80), AVSc (n = 78), and severe aortic stenosis (AS n = 80). A broad panel of circulating cytokines was measured and analyzed with respect to sex and disease stage. To assess the functional impact of key cytokines, *in vitro* experiments were conducted using human VECs and VICs treated with interleukin-1β (IL-1β) and interferon-β (IFNβ). Cellular responses were evaluated *via* morphological analyses, gene and protein expression assays, and calcification potential under normal and pro-osteogenic conditions.

**Results:**

Cytokine profiling revealed that AVSc patients exhibited significantly elevated levels of IL-1β compared to both CTRL and AS, with IL-1β being consistently higher in males across all stages. *In vitro*, IL-1β triggered endothelial-to-mesenchymal transition in VECs, promoting a pro-fibrotic and inflammatory phenotype. Sex-stratified analysis of VICs showed that IFNβ enhanced RUNX2 expression and calcification in a dose-dependent manner, with female-derived VICs being more responsive. Conversely, IFNβ exerted anti-fibrotic effects by reducing *COL1A1* and *ACTA2* expression, more markedly in female cells, particularly at the protein level.

**Conclusion:**

Our findings reveal a previously overlooked role of systemic inflammation, primarily driven by IL-1β and IFNβ, in promoting early endothelial activation and sex-specific fibrocalcific remodeling in FCAVD. These cytokines not only serve as markers of disease but also actively influence cell-specific responses, shaping the distinct aortic valve fibrocalcific patterns observed in men and women. Unraveling these mechanisms could open new avenues for developing early monitoring of circulating IL-1β and IFNβ, while informing sex-specific therapeutic strategies to modulate cytokine signaling to slow or prevent FCAVD progression.

## Introduction

Fibrocalcific aortic valve disease (FCAVD) is a multifactorial and progressive detrimental pathology that silently undermines the aortic valve function, affecting approximately 3% of individuals older than 65 years in the general population 1. The early stage of the disease, known as aortic valve sclerosis (AVSc), is marked by uneven thickening of the valve leaflets with calcium spots and no hemodynamic changes 2. However, in more than 10% of cases, this condition progresses to aortic valve stenosis (AS), where the narrowing of the valve creates significant resistance to blood flow and reduces the valve's functional area 3. Despite its high prevalence and associated cardiovascular morbidity and mortality 4, 5, there are currently no pharmacological therapies available to halt or even slow down FCAVD progression. To date, the only available treatments are surgical aortic valve replacement (AVR) or transcatheter aortic valve implantation (TAVI) 6, 7. However, these surgical approaches remain an incomplete solution, as the pathological mechanisms driving the disease persist, and the replaced valves are prone to degeneration over time. This highlights the urgent need to advance our understanding of the molecular mechanisms underpinning FCAVD, with the overarching goal of identifying potential therapeutic targets.

Although much remains to be understood about this condition, it is now known that FCAVD is an active process driven by multiple molecular pathways and different cell types, each of which plays a critical role in its onset and progression 8. Among the various mechanisms involved, local inflammation within the valvular structure is recognized as a key driver of disease onset and progression 9. However, most recently, attention has shifted to its role at a systemic level, exploring levels of circulating inflammatory markers and their role in FCAVD. In this regard, several studies have shown that stenotic patients exhibit elevated circulating cytokine levels compared to healthy controls, speculating that this systemic inflammatory increase could create a self-sustaining vicious cycle, further activating the earliest player in the disease process: valve endothelial cells (VEC) 10-12. Once damaged, these cells represent one of the primary upstream triggers of the complex cellular and molecular cascade underlying FCAVD 13. As the disease progresses, the initial inflammation subsides, paving the way for extensive extracellular matrix rearrangement that leads first to fibrosis and subsequently to calcification. This process, initiated by endothelial damage, is primarily driven by the myofibroblastic and osteoblastic differentiation of valvular interstitial cells (VIC), the other key cellular component of the aortic valve. In particular, VICs play a fundamental role not only in maintaining valvular integrity but also in driving disease progression, making them central players in the fibrocalcific remodeling characteristic of FCAVD 14, 15. Moreover, recent advances in understanding the pathophysiology of FCAVD have underscored the crucial role of sex-related differences in shaping key pathological processes related to fibrosis and calcification. In particular, it is now well established that men exhibit greater valvular calcification, whereas in women, the disease manifests primarily as increased fibrosis 16. Aortic valve transcriptomic analyses have confirmed sex-specific regulatory signatures, characterized by a prevailing pro-fibrotic remodeling response in women and an upregulation of pro-inflammatory, immune-associated pathways in men 8.

Despite these significant advancements in understanding the pathophysiology of FCAVD, little is known about the state of systemic inflammation during the early AVSc phase and how it may influence subsequent endothelial damage and the fibrocalcific remodeling of VICs. Moreover, given the well-established sex-specific differences in disease mechanisms, it remains unclear how these variations might further shape disease progression.

Thus, we hypothesize that systemic inflammation during the early stage of FCAVD, namely AVSc, exacerbates endothelial dysfunction and accelerates fibrocalcific remodeling, with potentially sex-different mechanisms. In line with this hypothesis, the present study aims to investigate the interplay between systemic inflammation, early endothelial dysfunction, and sex-specific fibrocalcific processes. Examining how systemic inflammatory mediators contribute to endothelial cell damage and VIC remodeling in the early stages of AVSc and exploring how these mechanisms differ between sexes may ultimately shed light on potential therapeutic strategies to halt or slow disease progression.

## Material and Methods

### Study population's enrollment

The study received approval from the Institutional Review Board and the Ethical Committee of Centro Cardiologico Monzino (CCM; R518/16-CCM547). The investigation was conducted in compliance with the Declaration of Helsinki (1964) and written informed consent was obtained from each participant. For the circulating cytokine evaluation, we enrolled healthy subjects from 2017 to 2022. Participants were excluded in the presence of a history of cardiac surgery, rheumatic heart disease, endocarditis, as well as active malignancy, chronic hepatic or renal disorders. Additional exclusion criteria included abnormalities in calcium regulation (including hyperparathyroidism, hyperthyroidism, and hypothyroidism), chronic or acute inflammatory conditions such as sepsis, autoimmune disease, inflammatory bowel disease, or the use of immunomodulating therapies, that might affect circulating cytokine levels. Each subject underwent echocardiographic evaluation for AVSc screening according to Stewart *et al*. criteria 17. In particular, AVSc was considered if a non-uniform thickening or spotty calcified areas of the aortic valve leaflets without significant hemodynamic changes (maximum aortic velocity < 2.5 m/s) was present. Whereas AS patients were enrolled, in the same period of time, when diagnosed at CCM and accordingly to the European Society of Cardiology (ESC) and the European Association for Cardio-Thoracic Surgery (EACTS) guidelines 18. All the subjects without any sign of aortic valve thickening or calcification were classified as controls (CTRL).

For the endothelial cell-based experiments, given the difficulty of obtaining healthy control valves, we used valves from patients undergoing AVR for severe aortic insufficiency, root aneurysm, or thoracic aortic aneurysm. Regarding the interstitial cell-based experiments, aortic valves were collected from patients undergoing AVR due to severe aortic valve stenosis.

### Blood sample collection and processing

Fasting peripheral blood samples were obtained from CTRL subjects and patients in Vacutainer Z tubes (Vacutainer Systems, Becton Dickinson, Franklin Lakes, NJ, USA; Cod: 367614). Following a 2-hour clotting period at 37°C in a thermostatically controlled bath, samples were centrifuged at 2000 rcf for 10 minutes at room temperature. Serum fractions were collected, aliquoted, and preserved at -80 °C pending analysis.

### Circulating cytokine evaluation

Circulating cytokine concentrations were measured using the human XL cytokine magnetic Luminex performance assay 44-plex fixed panel (Bio-techne, Minneapolis, MN, USA; Cod: LKTM014) according to the manufacturer's instructions. After thawing, serum samples were centrifuged at 16000 rcf for 5 minutes at 4C° and diluted 1:2 prior to analysis. Sample aliquots and standards were run in duplicate in the 96-well plate containing 50 µL of microparticles cocktail per well and incubated for 2 hours, at room temperature under continuous shaking (850 rpm). The plate was placed on a magnetic separator to retain the antibody-coated particles bound to target proteins, followed by three washes. Subsequently, 50 μL of diluted Biotin-Antibody Cocktail was added to each well and incubated for 1 hour at room temperature under uninterrupted shaking (850 rpm). After three additional washes, streptavidin was added and incubated for 30 minutes, at room temperature with constant shaking (850 rpm). Samples and standards were washed three times again, resuspended in wash buffer and read within 90 minutes using Bio-plex 200 system (BioRad, Hercules, CA, USA), setting the following acquisition protocol: a minimum of 50 beads per region, with 45-seconds per sample.

### Valve endothelial and interstitial cells isolation and treatments

Isolation of VECs from structurally healthy-looking aortic insufficient valves was performed using a modified method further developed by our group and described by Poggio *et al*. 19. Briefly, aortic valve leaflets were incubated in Advanced Dulbecco's modified Eagle's medium (AdDMEM -Life Technologies, Carlsbad, CA, USA; Cod: 12491023) containing 2 mg/mL type II collagenase (Worthington Biochemical Corp., Lakewood, NJ, USA; Cod: LS004176), 10% fetal bovine serum (FBS, Microtech, Naples, Italy; Cod: RM10432) and 1% penicillin-streptomycyn (Life Technologies, Carlsbad, CA, USA; Cod: 15070063). Following the incubation, leaflets were washed and endothelial were isolated using Dynabeads® conjugated with platelet endothelial cell adhesion molecule (CD31 - Life Technologies, Carlsbad, CA, USA; Cod: 11155D), an endothelial marker. Cells were then cultured in Medium 200 (M200 - Life Technologies, Carlsbad, CA, USA; Cod: M200500) supplemented with 10% FBS, 1% Penicilin-Streptomycyn and 2% of LSGS (Life Technologies, Carlsbad, CA, USA; Cod: S00310) on tissue culture plate coated with 0.1% gelatin (Sigma-Aldrich, St Louis, MO, USA; Cod: G1890). All the experiments with primary VECs were performed on cultured cells between their second and fifth passages, avoiding overgrowth or differentiation that could occur in later passages.

For stenotic VICs isolation after VEC isolation performed as above, the remaining specimens were carefully minced and treated with type II collagenase for four hours at 37°C to isolate VICs. After digestion of extracellular matrix components, VICs were seeded in tissue culture plates with complete Ad DMEM. After isolation, cells were cultured at 37°C in a 5% CO_2_ environment and subjected to immortalization 20.

Primary VECs were either left untreated or exposed daily to 10 ng/mL recombinant IL-1β (R&D System, Minneapolis, MN, USA; Cod: 201-LB-010) for three or five days, without media replacement. For calcium assay, male and female immortalized VICs (iVIC) were plated and treated every 2 days, with a change of medium at each interval, for a total of 7 days either in the presence or absence (normal media; NM) of pro-osteogenic media (POM; 10 mM β-glycerophosphate and 50 µg/mL ascorbic acid; BgAA), added after 1h of pre-treatment with or without IFNβ (Biotechne, Minneapolis, MN, USA; Cod: 8499-IF) at different concentrations (50, 500, and 1000 units/mL, U/mL; see **Supplementary Material and Methods** for rationale of concentration selection). For gene expression and immunofluorescence analyses, iVICs were pretreated with IFNβ at different concentrations for 1 hour, followed by treatment with POM or no treatment (NM) for 72 hours, after which the treatments were discontinued and the assays carried out.

### Reverse transcription and real-time PCR

Total RNA was isolated from primary VECs and iVICs with the Total RNA Purification Plus Kit (Norgen Biotek Corp., Thorold, ON, Canada; Cod: T2010S), according to the manufacturer's protocol. After gDNA removal, RNA purification and elution, the total quantity of RNA yield was quantified with Infinite® 200pro (TECAN) spectrophotometer. A total of 1000 ng RNA was retro-transcribed into cDNA using Luna® RT Supermix (New England Biolabs, Ipswich, MA, USA; Cod: E3010), following the manufacturer's protocol. Real-time PCR was performed on 25 ng of cDNA using Luna® Universal qPCR Master Mix (New England Biolabs, County Road Ipswich, MA, USA; Cod: M3003), following the manufacturer's instructions using gene-specific primers (primer sequences can be found in **Supplementary Table S1**).

### Calcification assay

At the end of 7 days, the media were collected and stored at -80 °C for subsequent measurement of secreted collagen. Extracellular calcium was dissolved using 0.6 M HCl, and after 5 hours of incubation under gentle shaking, the samples were collected and stored at +4 °C until analysis. Calcium levels in samples were then quantified using a calcium colorimetric assay kit (Waltham, MA, USA; Abcam, Cambridge, UK Cod: ab10250), following the manufacturer's protocol. For total protein quantification, cells were incubated overnight at +4 °C in 0.1 M NaOH containing 0.1% sodium dodecyl sulfate (SDS). Total proteins were subsequently measured using the bicinchoninic acid (BCA) Protein Assay (Thermo Fisher Scientific, Waltham, MA, USA; Cod:23227).

The absorbances of extracellular calcium and total protein quantification were measured using TECAN. The amount of calcium detected (in ng/µl) was normalized by dividing the total protein content (in µg/µl). Fold changes were then calculated.

### Immunofluorescence

Stenotic male and female iVICs were seeded in 96-well phenoplates (PerkinElmer; Cod: 6055300) in complete Ad DMEM medium. To evaluate αSMA levels, after 72 hours of treatments described in the above paragraph, the medium was removed and the cells were fixed in 4% formalin (Sigma-Aldrich; Cod: HT501128-4L, pH 6.9-7) for 15 min. Following two washes with 1× PBS, cells were incubated with 5 ug/mL Wheat Germ Agglutinin (WGA, ThermoFisher Scientific; w32464) for 10 minutes at room temperature. After one wash with 1× PBS, the cells were permeabilized with pre-cooled methanol (-20°C) for 10 minutes. Residual methanol was removed by two additional washes with 1× PBS (Merck Life Science S.R.L.; Cat. 322415), and a blocking solution containing 5% BSA (Sigma-Aldrich; Cat. 05482-25G), 0.5% donkey serum (Sigma-Aldrich; Cat. D9663), and 0.2% Triton X-100 (Merck Life Science S.R.L.; Cat. X100-100ML) was added to the wells for 30 minutes at room temperature to prevent non-specific binding. After two washes in 1× PBS, cells were then incubated overnight in blocking solution with primary antibodies against α-SMA (abcam; Cod: ab5694). Detection was performed using anti-rabbit Alexa Fluor 488 secondary antibody (Jackson ImmunoResearch Laboratory; Cod: 711-545-152). Nuclei were counterstained with Vectashield Mounting Medium containing DAPI (D.b.a Italia S.r.l.; Cod: H-1200-10). Images were acquired using a high-throughput microplate confocal imager for high-content screening (Operetta CLS, PerkinElmer), and analysis was conducted with Harmony software (version 5.1).

### Enzyme-linked immunosorbent assay

The secreted pro-collagen 1a1 (ProCol1a1) protein was measured in the supernatant of iVICs after treatment using a commercial enzyme-linked immunosorbent assay (ELISA) kit (R&D Systems, Minneapolis, MN, USA; Cod: DY6220), following the manufacturer's instructions. Each sample was diluted 1:1000 and the amount of ProCol1a1 obtained in pg/µL was normalized to the total protein quantification in µg/µL, resulting in the calculation of pg of ProCol1a1 per µg of protein.

### Statistical analysis

Statistical analyses were conducted using GraphPad Prism (version 10.4.1, GraphPad Software, San Diego, CA, USA) and R software (version 4.2.2). Continuous variables were reported as median and interquartile range (IQR). Differences among study groups (male and female) were analyzed using one-way ANOVA with Bonferroni post-hoc correction. Dose-response effects were tested with linear regression trend analysis. Differences between study groups were analyzed using two-way ANOVA with interaction and partial eta-squared (η^2^_p_) was used for the effect size evaluation. The power calculation was performed based on the number of independent datapoints per group (n = 6, across 8 experimental groups). Assuming a standard deviation of 0.2, a Bonferroni-adjusted two-tailed significance level of 0.0167, and a desired power of 0.80, the minimum detectable log2 fold change (Log2FC) was 0.438. Statistical significance was defined as a two-tailed pValue < 0.05 after Bonferroni correction when appropriate. The effect size was defined as 0.01 ≤ small effect ≤ 0.06; 0.06 < medium effect ≤ 0.10; large effect > 0.10, according to Maher JM et. al. 21.

## Results

### Patients' characteristics

A total of 238 subjects were enrolled in the study: 80 subjects with no signs of aortic valve thickening or spotty calcification (CTRL), 78 subjects with aortic valve leaflet thickening and spotty calcification (AVSc), and 80 patients with severe aortic valve stenosis (AS). The groups were comparable for age, sex, prevalence of diabetes, smoking habits, and left ventricular ejection fraction (LVEF). A slight increase in BMI was observed between the CTRL and AVSc groups, which was significant when comparing AVSc and AS patients. As expected, peak aortic jet velocity increased progressively with the pathology progression (**Table 1**).

### Elevated systemic inflammation in the AVSc stage

**Figure 1:**
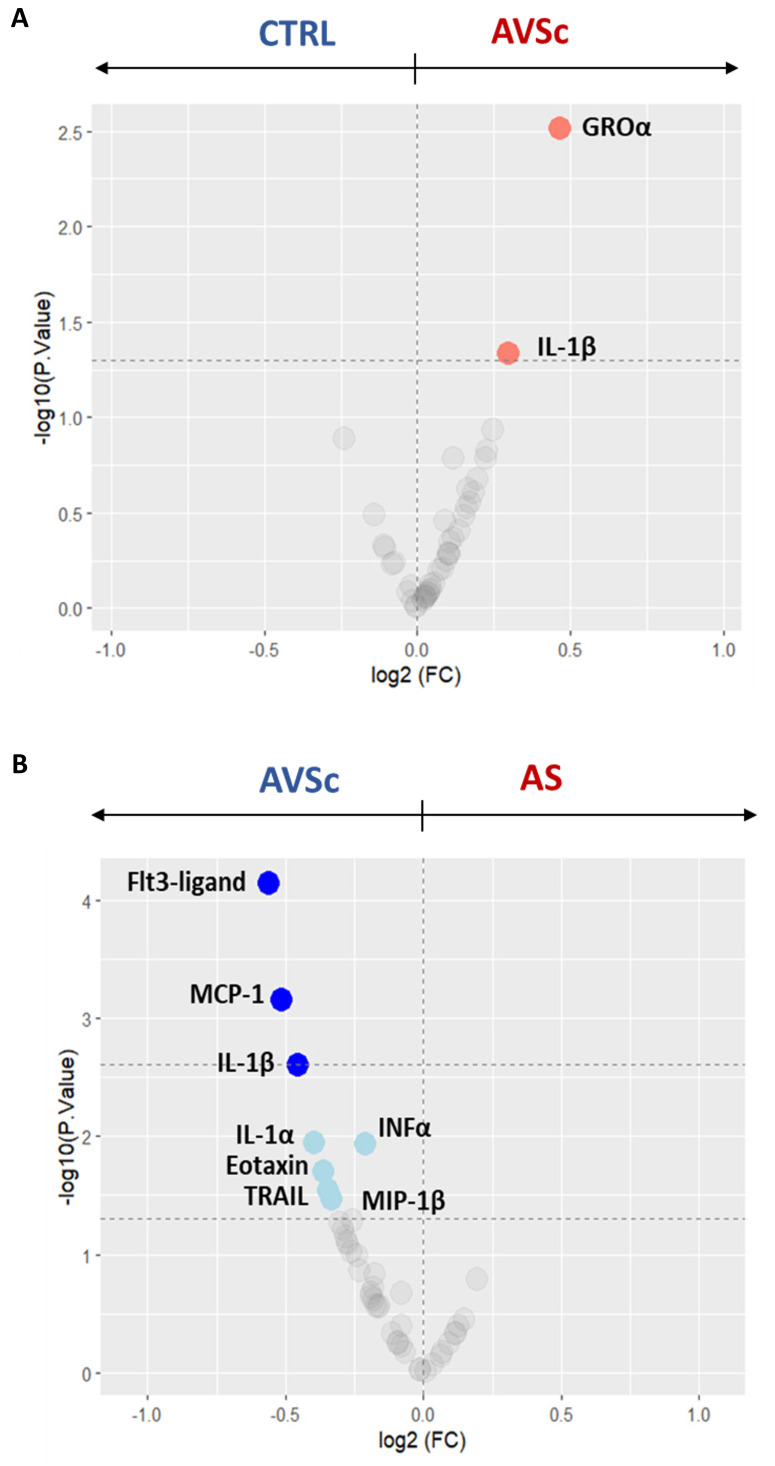
**Serum cytokine profiling across the stages of fibrocalcific aortic valve disease (FCAVD).** Volcano plots showing the differential levels of circulating cytokines of (**A**) aortic valve sclerosis subjects (AVSc; n = 78) in respect to control subjects (CTRL; n = 80) and (**B**) AVSc subjects in respect to severe aortic stenosis (AS; n = 80) patients. Coloured dots indicate the cytokine differentially expressed. Light colour indicates cytokines with a pValue < 0.05 (dashed line at -log10 of 1.3), while dark colour indicates a pValue < 0.002 (dashed line at -log10 of 2.7).

Circulating cytokines across different stages of the pathology were measured to gain deeper insight into the systemic inflammatory dynamics. To specifically investigate inflammatory changes associated with disease progression, analyses in this section were primarily focused on comparisons involving the AVSc stage. Serum cytokine profiling revealed consistently elevated inflammation levels in AVSc subjects, compared to both CTRL and AS subjects. Specifically, when compared to CTRL, an up-regulation of growth-regulated oncogene-α (GROα; p = 0.003) and IL-1β (p = 0.046) was observed (**Figure 1A** and **Supplementary Table S2**). While, when compared to AS, AVSc cases exhibited significantly higher levels of FLT3-ligand (p < 0.0001), MCP1 (p = 0.001), IL1β (p = 0.002), interleukin-1 α (IL-1α; p = 0.011), interferon- α (IFNα; p = 0.012), eotaxin (p = 0.020), tumor necrosis factor-related apoptosis-inducing ligand (TRAIL; p = 0.029), and macrophage inflammatory protein-1β (MIP-1β; p = 0.034) (**Figure 1B** and **Supplementary Table S2**). Nevertheless, AS patients also showed an alteration in the inflammatory profile marked by elevated tumor necrosis factor (TNFα; p = 0.03) alongside a down-regulation of several key chemokines and growth factors, including motif chemokine ligand 1 (MCP1; p < 0.0001), fms-related tyrosine kinase 3 ligand (FLT3-ligand; p = 0.004), eotaxin (p = 0.03), and epidermal growth factor (EGF; p = 0.03) compared to CTRL subjects (**Supplementary Figure S1** and **Supplementary Table S2**).

### IL-1β drives endothelial to mesenchymal transition of aortic valve endothelial cells

**Figure 2:**
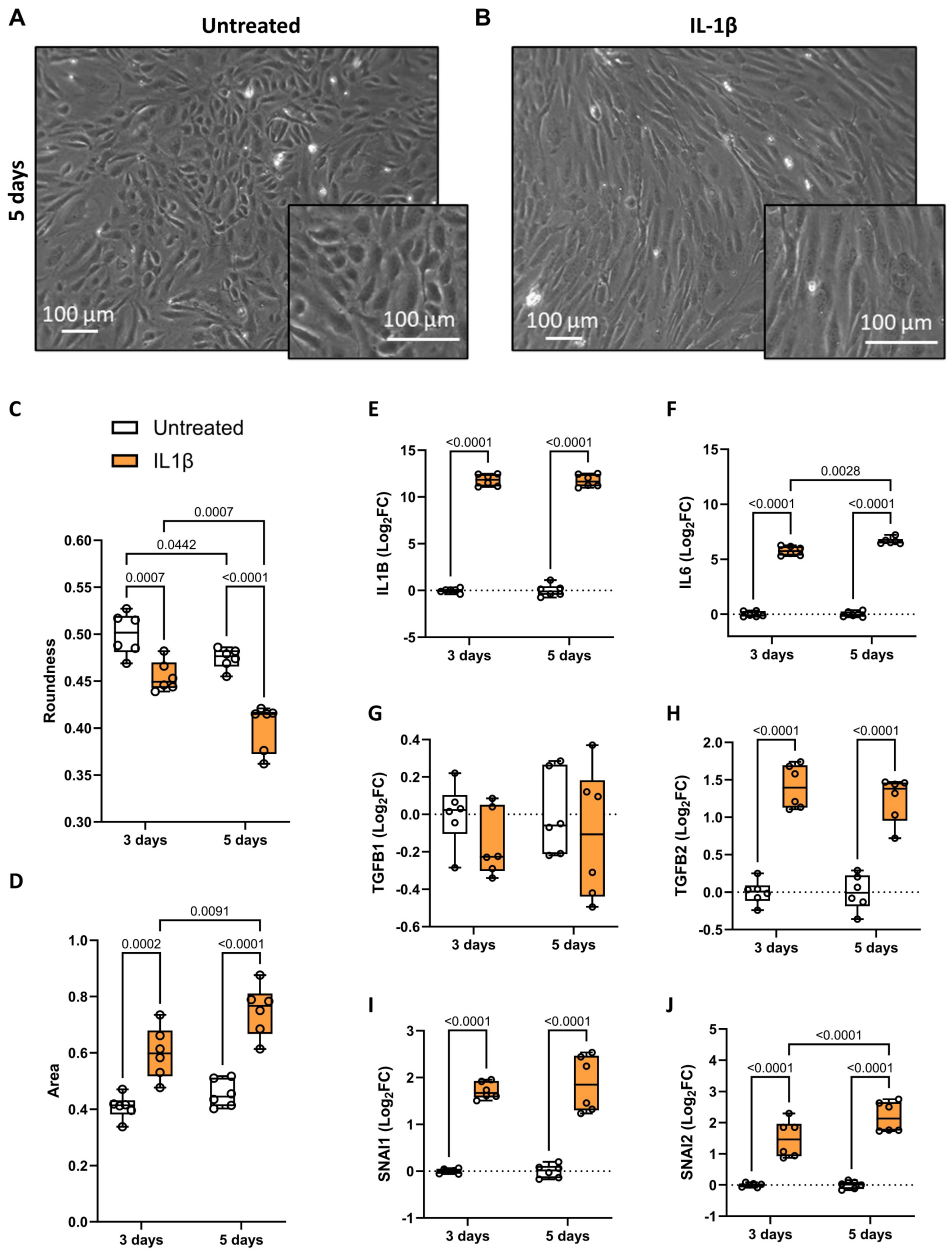
** Valve endothelial cells response to IL-1β treatment.** Representative brightfield images showing valve endothelial cells (VEC), after 5 days in culture, (**A**) in the absence of treatment and (**B**) after treatment with 10 ng/mL IL-1β. Magnification 20x; Scale bar: 100 µM. Box and whisker plots representing the roundness (**C**) and area (**D**) of untreated (white) and IL-1β-treated (orange) VECs after three and five days in culture. Each data point represents the average of 1.096 ± 104 cells. Box and whisker plots showing gene expression of (**E**) *IL1B*, (**F**) *IL6*, (**G**) *TGFB1*, (**H**) *TGFB2*, (**J**) *SNAI1*, and (**K**) *SNAI2* in untreated (white; n = 12) and IL-1β-treated (orange; n = 12) VECs after three and five days in culture. Data are presented as median ± interquartile range with minimum and maximum values. For the experiments, primary VECs derived from two independent donors (one male and one female) were used. For each donor, we performed three independent experiments, yielding a total of six independent experimental replicates per condition (n = 6). All represented pValues are calculated by two-way ANOVA and the effect sizes are reported in **Supplementary Table S3**.

To explore the role of up-regulated inflammatory status in AVSc, we focused on IL-1β. Considering its well-described ability to induce endothelial-to-mesenchymal transition (EndMT) 22, 23, we treated control VECs with recombinant IL-1β to investigate its effect on valve endothelium in the context of FCAVD. Analyzing cell morphology, untreated VECs displayed their characteristic cobblestone shape, which shifted to a more elongated form after IL-1β treatment (**Figure 2A**, **2B**, and **Supplementary Figure S2**), suggesting an EndMT onset. To validate these observational data and to confirm the phenotypic change in VECs, we assessed specific parameters such as cell roundness and area. At both time points after IL-1β treatment, we observed a significant reduction in roundness (**Figure 2C** and **Supplementary Table S3**), along with an increase in cell area (**Figure 2D** and **Supplementary Table S3**), confirming the morphological changes in the valve endothelium following a pro-inflammatory stimulus. To further investigate IL-1β-mediated activation of VECs, we assessed the gene expression of *IL1B*, *IL6*, *TGFB1*, *TGFB2*, and *SNAI1/2*. IL-1β treatment significantly up-regulated all these genes but *TGFB1*, with IL6 and SNAI2 showing time-dependent increases, confirming the activation of inflammatory, mesenchymal transformation, and pro-fibrotic pathways (**Figure 2E-J** and **Supplementary Table S3**).

### Sex-related divergence in cytokine profiles during disease progression

Given the sex-dependent characteristics of FCAVD 24, we further analyzed the circulating cytokine levels by stratifying patients according to sex.

**Figure 3:**
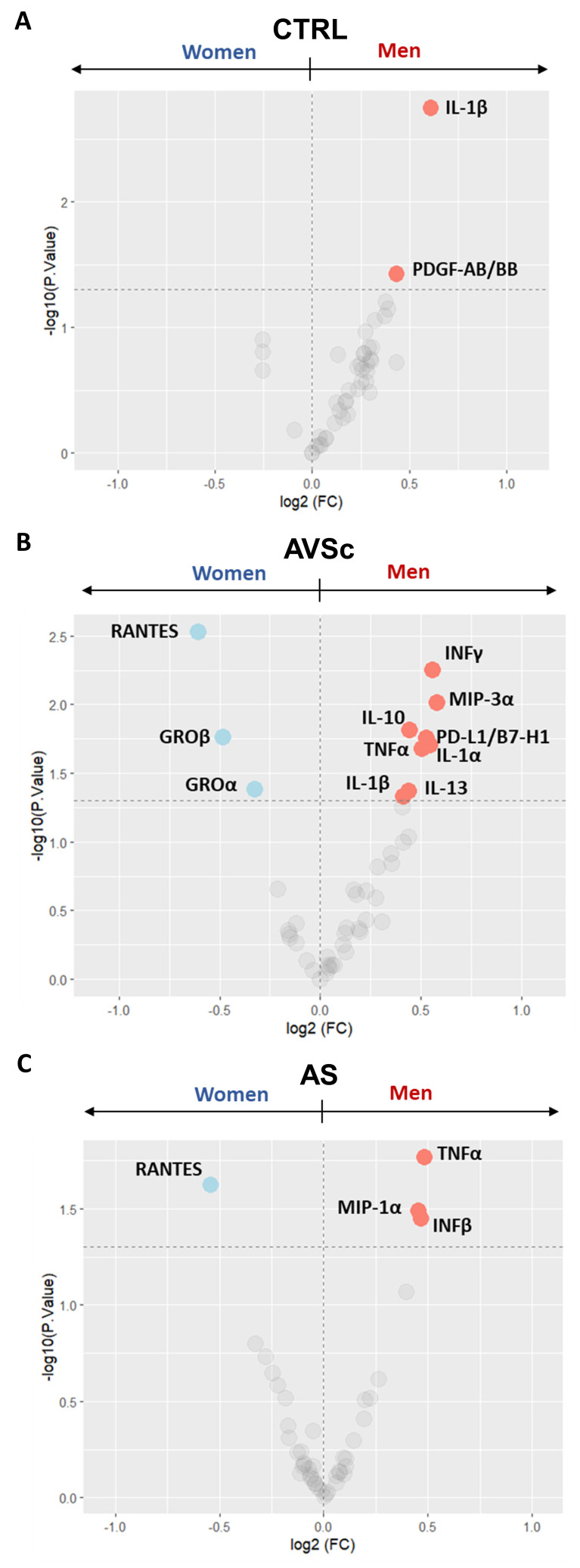
**Sex-stratified serum cytokine profiling across fibrocalcific aortic valve disease (FCAVD) stages.** Volcano plots showing the differential levels of circulating cytokines in (**A**) control (CTRL; 40 men and 40 women) subjects, (**B**) aortic valve sclerosis (AVSc; 39 men and 39 women) subjects, and (**C**) severe aortic stenosis (AS; 48 men and 32 women) patients, focusing on sex difference in each group (men *vs.* women). Light coloured dots indicate the cytokine differentially expressed (pValue < 0.05; dashed line at -log10 of 1.3).

We observed that control men exhibited higher levels of IL-1β (p = 0.002) and platelet-derived growth factor AB/BB (PDGF-AB/BB; p = 0.037) when compared to control women (**Figure 3A** and **Supplementary Table S4**), although the IL-1β levels remained within the normal range (all but one ≤ 12 pg/mL; **Supplementary Figure S3**) 25. As the disease progresses through its stages, this male-specific trend of elevated circulating inflammatory cytokines persists. Indeed, in AVSc group, men showed higher levels of interferon-γ (INFγ; p = 0.006), macrophage Inflammatory Protein-3 (MIP3α; p = 0.010), programmed cell death 1 ligand 1 (PD-L1/B7-H1; p = 0.020), IL-1β (p = 0.046), tumor necrosis factor-α (TNFα; p = 0.015) and IL-1α (p = 0.021), along with increased anti-inflammatory cytokines Interleukin-10 (IL-10; p = 0.017) and Interleukin-13 (IL-13; p = 0.042). Conversely, they exhibited lower levels of GROα (p = 0.041), growth-regulated oncogene-β (GROβ; p = 0.017), and regulated upon activation, normally t cell expressed and presumably secreted (RANTES; p = 0.003) compared to AVSc women (**Figure 3B** and **Supplementary Table S4**). Finally, in the advanced stages of the disease, men with AS consistently showed higher amounts of TNFα (p = 0.017), MIP1α (p = 0.036), and INFβ (p = 0.032), with a lower amount of RANTES (p = 0.024) than their women counterparts (**Figure 3C** and **Supplementary Table S4**).

### INFβ drives sex-specific calcification in stenotic VICs

Prompted by the sex-specific inflammatory profiles observed across disease stages, we next investigated the effects of IFNβ on VIC calcification. As expected, female iVICs exhibited significantly lower calcification levels compared to those from male patients at steady state and under POM conditions (**Supplementary Figure S4A** and **S4D**).

**Figure 4:**
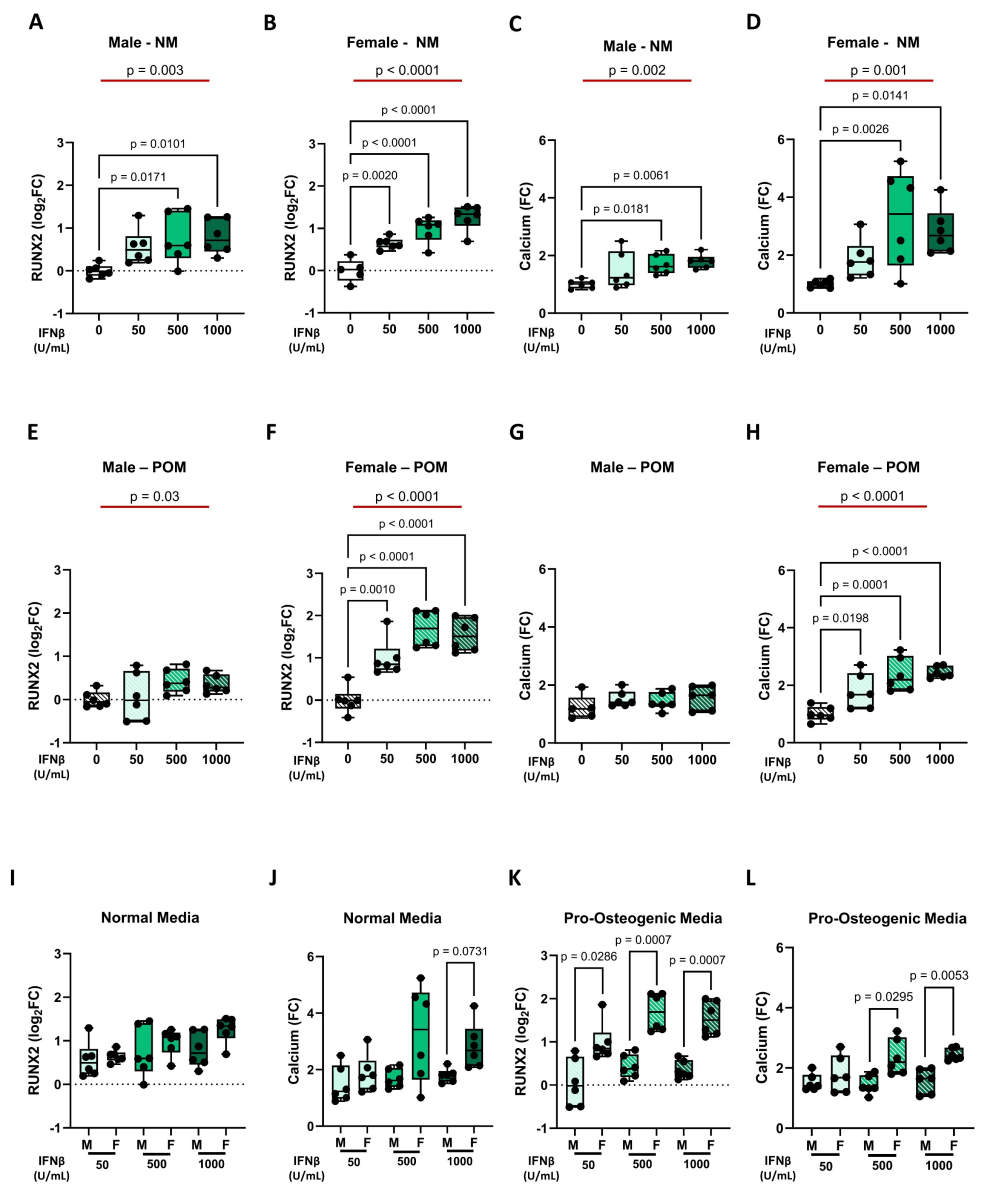
** Impact of INFβ on *RUNX2* expression and calcification in male and female iVICs.** Box and whisker plots representing the gene expression level of *RUNX2* in (**A**) male and (**B**) female iVICs cultured in normal media (NM) supplemented with different concentrations of INFβ (0, 50, 500, and 1000 U/mL). Box and whisker plots representing the calcium deposition in (**C**) male and (**D**) female iVICs cultured in NM supplemented with different concentrations of INFβ. Box and whisker plots representing the gene expression level of *RUNX2* in (**E**) male and (**F**) female iVICs cultured in pro-osteogenic media (POM) supplemented with different concentrations of INFβ. Bar graphs representing the calcium deposition in (**G**) male and (**H**) female iVICs cultured in POM supplemented with different concentrations of INFβ. Box and whisker plots representing the comparison of (**I**) *RUNX2* and (**J**) calcium deposition between men and women iVICs treated with different concentrations of INFβ in NM. Box and whisker plots representing the comparison of (**K**) *RUNX2* and (**L**) calcium deposition between men and women iVICs treated with different concentrations of INFβ in POM. Data are presented as median ± interquartile range with minimum and maximum values. For the experiments, immortalized VICs derived from independent human donors (6 males and 6 females) were used. For each donor line, measurements were obtained under the specified experimental conditions; technical replicates (when performed) were aggregated within donor before statistical testing so that the donor remained the unit of inference. (**A-H**) The represented pValues on black lines are one-way ANOVA post-hoc test with Bonferroni correction and the represented pValue on the red line is the test for trend. (**I-L**) The presented pValue are calculated by two-way ANOVA and the effect sizes are reported in **Supplementary Table S5**.

Regarding IFNβ effects, we obtained similar results at both gene expression level (*e.g., RUNX2*) and functional level, as indicated by the calcification potential. In NM condition, IFNβ treatment led to a dose-dependent increase in *RUNX2* expression and calcification potential, both in male iVICs (**Figure 4A** and** 4C**) and female iVICs (**Figure 4B** and **4D**). Similarly, under POM influence, INFβ was also able to exacerbate the up-regulation of *RUNX2* and calcification, but to a lesser extent in male iVICs (**Figure 4E** and **4G**) compared to female iVICs (**Figure 4F** and **4H**). Indeed, the comparison between male and female iVICs, both at the gene and functional level, revealed that the former ones were significantly less susceptible to INFβ (**Figure 4I-L** and **Supplementary Table S5**).

### INFβ-mediated suppression of fibrosis exhibits sexual dimorphism

We next evaluated the effects of INFβ fibrosis by analyzing *COL1A1* gene and protein expression in male and female iVICs. As expected, without INFβ treatment, female iVICs exhibited higher collagen production than male patients under NM and POM conditions (**Supplementary Figure S4B** and **S4E**).

**Figure 5:**
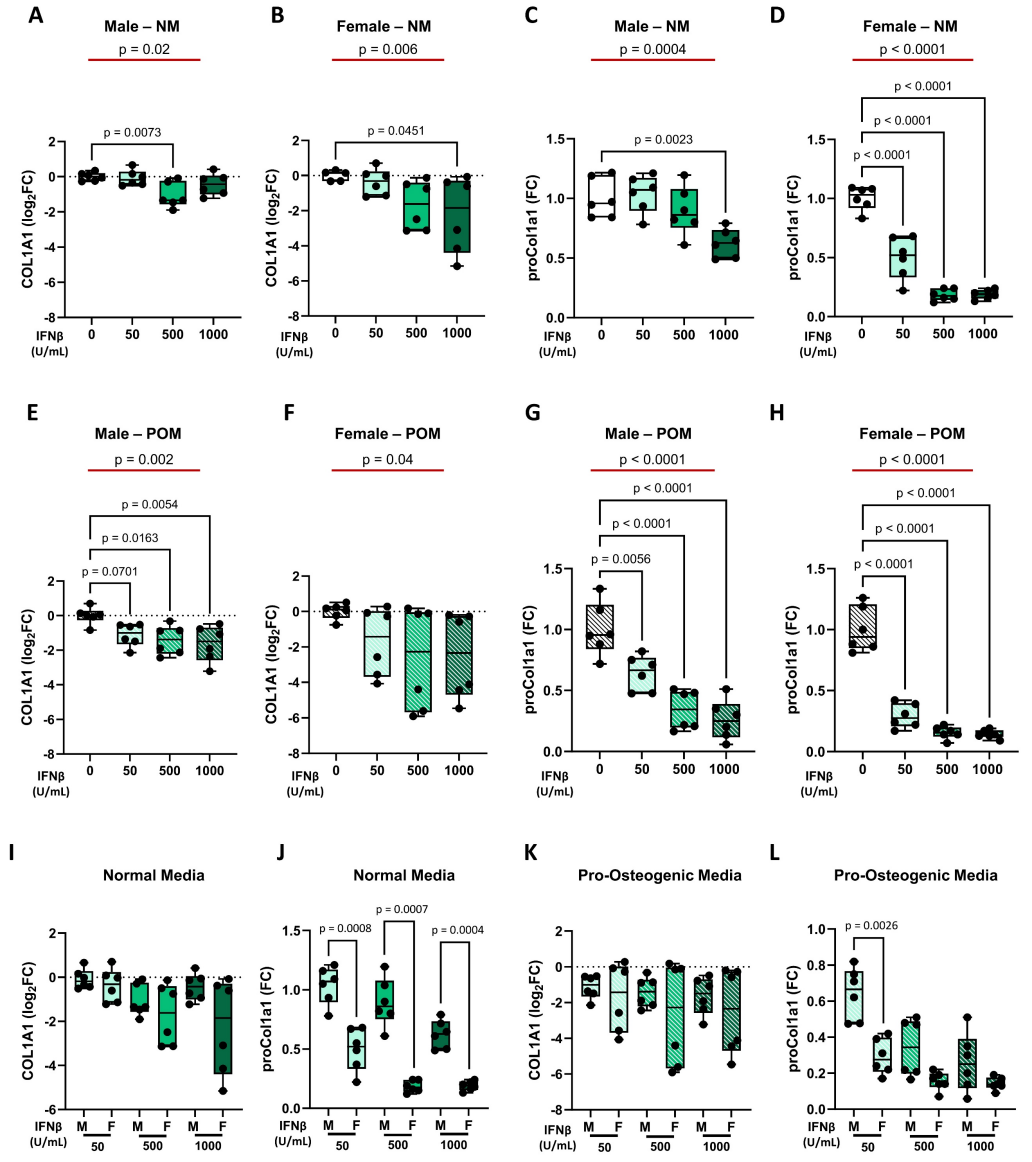
** Regulation of collagen expression by IFNβ at mRNA (*COL1A1*) and protein (proCol1A1) levels in male and female iVICs.** Box and whisker plots representing the gene expression level of *COL1A1* in (**A**) male and (**B**) female iVICs cultured in normal media (NM) supplemented with different concentrations of INFβ (0, 50, 500, and 1000 U/mL). Box and whisker plots representing proCol1a1 expression in (**C**) male and (**D**) female iVICs cultured in NM supplemented with different concentrations of INFβ. Box and whisker plots representing the gene expression level of *COL1A1* in (**E**) male and (**F**) female iVICs cultured in pro-osteogenic media (POM) supplemented with different concentrations of INFβ. Box and whisker plots representing proCol1a1 expression in (**G**) male and (**H**) female iVICs cultured in POM supplemented with different concentrations of INFβ. Box and whisker plots representing the comparison of (**I**) *COL1A1* and (**J**) proCol1a1 protein expression between men and women treated with different concentrations of INFβ in NM. Box and whisker plots representing the comparison of (**K**) *COL1A1* and (**L**) proCol1a1 expression between men and women treated with different concentrations of INFβ in POM. Data are presented as median ± interquartile range with minimum and maximum values. For the experiments, immortalized VICs derived from independent human donors (6 males and 6 females) were used. For each donor line, measurements were obtained under the specified experimental conditions; technical replicates (when performed) were aggregated within donor before statistical testing so that the donor remained the unit of inference. (**A-H**) The represented pValues on black lines are one-way ANOVA post-hoc test with Bonferroni correction and the represented pValue on the red line is the test for trend. (**I-L**) The presented pValue are calculated by two-way ANOVA and the effect sizes are reported in **Supplementary Table S6**.

Regarding IFNβ effects on collagen production, it was similar at both the gene and protein levels. In particular, in NM, IFNβ treatment led to a dose-dependent decrease of collagen, with a weaker effect in male iVICs (**Figure 5A** and **5C**) compared to female iVICs (**Figure 5B** and **5D**). Similarly, under POM influence, INFβ reduced collagen expression at gene and protein levels, both in male iVICs (**Figure 5E** and **5G**) and in female iVICs (**Figure 5F** and **5H**). Direct comparison between male and female iVICs confirmed a lower susceptibility of male iVICs to IFNβ at the protein level (**Figure 5I-L** and **Supplementary Table S6**).

### INFβ interferes with the myofibroblastic phenotype of VICs in a sex-dependent manner

The effects of IFNβ on VIC myofibroblastic activation were evaluated by analyzing *ACTA2* gene expression and αSMA protein levels. Opposite to collagen production, female iVICs exhibited lower *α*SMA levels than male iVICs at steady state and under POM conditions (**Supplementary Figure S4C** and **S4F**).

**Figure 6:**
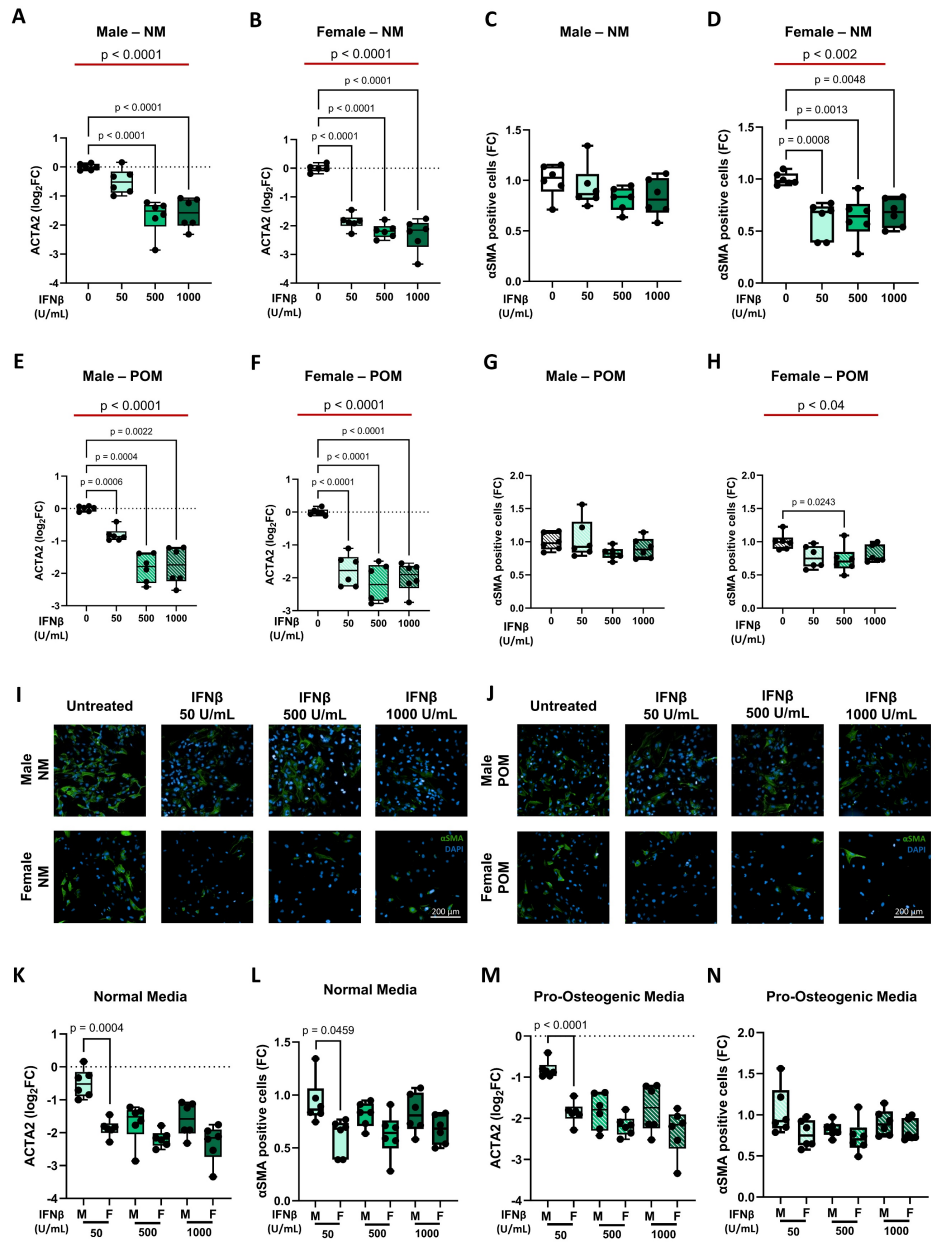
** Influence of IFNβ on smooth muscle actin expression at mRNA (*ACTA2*) and protein (αSMA) levels in male and female iVICs.** Box and whisker plots representing the gene expression level of *ACTA2* in (**A**) male and (**B**) female iVICs cultured in normal media (NM) supplemented with different concentrations of INFβ (0, 50, 500, and 1000 U/mL). Box and whisker plots representing αSMA expression in (**C**) male and (**D**) female iVICs cultured in NM supplemented with different concentrations of INFβ. Bar graphs representing the gene expression level of *ACTA2* in (**E**) male and (**F**) female iVICs cultured in pro-osteogenic media (POM) supplemented with different concentrations of INFβ. Box and whisker plots representing αSMA expression in (**G**) male and (**H**) female iVICs cultured in POM supplemented with different concentrations of INFβ. Representative immunofluorescence images showing αSMA (green) expression and localization in male (upper panels) and female (lower panels) iVICs cultured in (**I**) NM and (**J**) POM supplemented with different concentrations of INFβ. Nuclei were visualized with 4',6-diamidino-2-fenilindolo (DAPI; blue). Magnification 20x. Scale bar: 200 µM. Box and whisker plots representing the comparison of (**K**) *ACTA2* and (**L**) αSMA expression between men and women iVICs treated with different concentrations of INFβ in NM. Box and whisker plots representing the comparison of (**M**) *ACTA2* and (**N**) αSMA expression between men and women iVICs treated with different concentrations of INFβ in POM. Data are presented as median ± interquartile range with minimum and maximum values. For the experiments, immortalized VICs derived from independent human donors (6 males and 6 females) were used. For each donor line, measurements were obtained under the specified experimental conditions; technical replicates (when performed) were aggregated within donor before statistical testing so that the donor remained the unit of inference. (**A-H**) The represented pValues on black lines are one-way ANOVA post-hoc test with Bonferroni correction and the represented pValue on the red line is the test for trend. (**K-N**) The presented pValue are calculated by two-way ANOVA and the effect sizes are reported in **Supplementary Table S7**.

In line with collagen data, under NM conditions, IFNβ treatment led to a dose-dependent down-regulation of *ACTA2*, at the transcriptional level (**Figure 6A** and **6B**). At protein levels, a pronounced reduction was observed only in female-derived cells (**Figure 6C** and **6D** and **Figure 6I**; **Supplementary Figure S6** and **S7**). Under POM, IFNβ similarly reduced *ACTA2* transcription in both sexes (**Figure 6E** and **6F**), while the reduction in αSMA protein levels remained more marked in female cells (**Figure 6G** and **6H** and **Figure 6J**;** Supplementary Figure S5** and **S6**). Direct comparison between male and female iVICs revealed a more pronounced reduction in *ACTA2* gene expression and αSMA protein levels in females, particularly at 50 U/mL IFNβ concentration, under NM and POM conditions (**Figure 6K-N** and **Supplementary Table S7**).

## Discussion

Building upon the recognized complexity of fibrocalcific aortic valve disease (FCAVD), our study provides novel insights into the interplay between systemic inflammation, endothelial dysfunction, and sex-related differences that characterize the early and progressive stages of the disease. Through a combination of patient profiling and *in vitro* modeling, we identify IL-1β and IFNβ as key modulators of endothelial activation, mesenchymal transformation, extracellular matrix remodeling, and calcification, with distinct sex-specific responses.

While previous studies have emphasized the pivotal role of the local inflammation and cellular transdifferentiation within the aortic valve leaflets 26-29, the contribution of systemic inflammation mediators remains underexplored and undervalued.

Prior investigations focusing on C-reactive protein (CRP), a common marker of systemic inflammation, have yielded conflicting results, with most finding no significant association between CRP levels and either AVSc presence or AS severity 30-32, although one study conducted on a smaller cohort suggested a possible link 33. However, the study did not provide sufficient evidence to use high-sensitive CRP (Hs-CRP) as a marker of disease progression, even if it did suggest that Hs-CRP may help identify patients with early-stage FCAVD who could benefit from treatment to prevent irreversible valve damage. Moreover, another recent study identified the systemic immune-inflammation index (SII), a composite marker based on neutrophil, lymphocyte, and platelet counts, as an independent predictor of severe calcific AS, but its application is limited to advanced stages of the disease, offering little insight into early inflammatory changes 34. Taken together, these observations highlight the need to look beyond traditional inflammatory markers and begin to consider circulating inflammatory factors as active contributors to FCAVD progression. In support of this, recent serum proteomic studies identified annexin A2 and cystatin C as mediators of sex-specific myofibroblast activation and osteoblast-like differentiation in VICs 35, while valvular proteomics revealed stage- and sex-specific protein signatures that parallel tissue remodeling patterns 36, further underscoring an active role of the systemic compartment in disease progression rather than a passive epiphenomenon of FCAVD. In this context, our findings extend these observations, showing that AS patients exhibit elevated TNFα alongside a broader reshaping of chemokine and growth factor networks. Moreover, in line with this, our study screened specifically a broad panel of circulating cytokines across all different stages of pathology with a particular focus on the early AVSc phase. In this stage, we identified elevated levels of circulating IL-1β and GROα, extending previous observations by linking systemic cytokine profiles directly to early valvular remodeling and offering mechanistic insight that clarifies the role of subclinical inflammation in shaping disease progression. Notably, although IL-1β levels remained within physiological ranges 25, their relative increase alongside GROα suggests a subtle, subclinical systemic inflammatory state during the early phase of the disease. Even these modest elevations may be sufficient to sensitize the valvular endothelium, promoting endothelial-to-mesenchymal transition (EndMT) and priming pro-fibrotic pathways, as reflected by the *IL6*, *SNAI2*, and *TGFB2* upregulation observed *in vitro*, suggesting an auto-amplifying inflammatory loop that could initiate early valve remodeling. Thus, this condition, while not reaching overt inflammatory thresholds, may still be sufficient to trigger the onset of the disease, playing a key role as in other cardiovascular pathologies 37. Moreover, pro-inflammatory cytokines have been increasingly implicated in the onset and progression of EndMT, myofibroblastic activation, and calcification within the aortic valve 23, 38-41. Building on these findings, functionally our study showed that the modest yet consistent increase in circulating IL-1β observed in AVSc patients could not be merely a biomarker but could play an active role in driving early endothelial dysfunction through its direct role on endothelial cells covering the aortic valve leaflets. Indeed, in line with prior studies using vascular and retinal endothelial cell models 23, 42, 43, we showed that IL-1β directly induces EndMT in human VECs, supporting its possible involvement in the early stage of FCAVD, acting as a potential upstream trigger of endothelial transformation, one of the first recognized triggers of the FCAVD 28.

In addition to its direct effect on VECs, IL-1β displayed distinct sex-related systemic patterns, with men consistently showing higher levels in the early disease stage (*i.e.*, AVSc) and even in healthy controls. This male-biased elevation may predispose male valves to earlier calcification, in line with the greater calcium burden observed clinically in men 8, 44, 45. In fact, supporting this, IL-1β is known to promote VICs mineralization 46 while inhibiting myofibroblastic activation 47, and in synergy with TNFα and IL-6, it can shift VICs from a myofibroblast phenotype to an osteoblastic-like one 48. Therefore, our study contributes to the understanding of FCAVD pathophysiology by showing how systemic IL-1β early orchestrates endothelial dysfunction and VIC remodeling, further strengthening the link between subclinical inflammation and sex-specific remodeling patterns.

IFNγ, another inflammatory cytokine elevated in men with AVSc, appears to exert this calcific push more effectively in male-derived VICs 49, potentially contributing to the greater calcification and lower fibrosis observed in men. In the later stages of FCAVD, our data highlight IFNβ as a new potential player able to modulate sex-specific valvular remodeling. Its systemic upregulation in men with AS coincides with a dual biological role: promoting calcification while suppressing myofibroblastic activation and fibrosis. Of note, while male-derived VICs are intrinsically more prone to calcify, IFNβ exerted a stronger pro-osteogenic and anti-fibrotic effect on female-derived VICs, suggesting that women may be more responsive to this cytokine's signaling.

The intrinsic role of these cytokines may explain the sexual dimorphism observed in AS patients, given that men's aortic valves have a greater calcium burden and lower fibrotic content compared to women's valves 16, 24. These data are also in accordance with clinical data showing a higher prevalence of FCAVD in men 50, as well as imaging and biological studies revealing a greater calcium load and reduced fibrotic tissue in male aortic valves 8, 24, 44, 45, 51, 52. Taken together, our findings suggest that sex-specific systemic cytokine environments may shape the trajectory of FCAVD toward distinct remodeling pattern, favoring fibrosis or calcification. This provides a potential mechanistic explanation for the sexual dimorphism observed in AS, highlighting how systemic inflammatory cues may interact with intrinsic cellular responses to direct valve remodeling.

Beyond merely reflecting disease presence, these cytokine imbalances, particularly involving IL-1β and IFNβ, appear to actively drive the cellular processes that initiate and sustain FCAVD. By linking systemic inflammation to early endothelial dysfunction and valvular interstitial cell remodeling, our data reveal a dynamic, sex-specific interplay that determines the progression of valve pathology. Importantly, this emerging paradigm suggests that even subtle, subclinical shifts in systemic cytokines may have outsized effects on disease trajectory, priming valves for either calcific or fibrotic outcomes. Recognizing these sex-specific systemic cues not only deepens our understanding of FCAVD pathophysiology but also opens new avenues for precision interventions based on patient stratification. Targeting key cytokine pathways, such as IFNβ, could allow for sex-tailored therapeutic strategies aimed at modulating disease progression from its earliest stages, potentially preventing irreversible valve damage.

Crucially, our study underscores a shift from viewing systemic inflammation as a passive biomarker toward recognizing it as an active, sex-specific orchestrator of valve remodeling, potentially redefining how we conceptualize and ultimately target FCAVD.

Despite the significant findings of this study, a few limitations need to be considered. This is a single-center study, which may limit the generalizability of the findings to broader AVSc and AS populations. Longitudinal follow-up data from our cohort are not available. Thus, future prospective studies are needed to evaluate the temporal dynamics of circulating pro-inflammatory cytokines and to substantiate their causal role in the progression of FCAVD. Selection bias may have been introduced through our eligibility criteria, which could have influenced the observed cytokine distributions and the possibility of residual confounding could persist due to unmeasured variables. Additionally, VECs used as controls were isolated from aortic valves of patients with valve insufficiency rather than from completely healthy tissue. Although this tissue is not entirely healthy, it does not present the typical fibrosis and calcification characteristic of stenotic valves. Therefore, while these cells may not fully reflect the characteristics of healthy VECs, they offer a reasonable compromise for studying aortic valve biology 9. Similarly, the use of iVICs, which provide a reliable and reproducible model, may not fully capture the complexity of primary VIC behavior. While they overcome practical limitations such as senescence and variability in primary cells, immortalized VICs cannot entirely replicate the physiological dynamics of native VICs.

## Supplementary Material

Supplementary figures and tables.

## Figures and Tables

**Table 1:** Patients' characteristics.

	CTRL (n=80)	AVSc (n=78)	AS (n=80)	ANOVA pValue
**Sex (male)**	40 (50%)	39 (50%)	48 (60%)	0.25
**Age (years)**	63.5±4.2	64.1±5.8	62.5±6.8	0.27
**Hypertension**	29 (36.3%)	42 (53.8%) *	58 (72.5%) ^#^	**< 0.0001**
**Dyslipidemia**	15 (18.8%)	27 (34.6%) *	29 (36.3%)	**0.009**
**Diabetes**	4 (5.0%)	9 (11.5%)	9 (11.3%)	0.18
**Smoking**	8 (10.0%)	15 (19.2%)	9 (11.3%)	0.55
**BMI (kg/m^2^)**	24.6±4.1	25.8±4.5	28.1±5.2 ^##^	**< 0.0001**
**LVEF (%)**	61.4±5.7	61.5±9.5	62.9±7.7	0.29
**Peak jet velocity (m/s)**	1.3±0.2	1.4±0.3 *	4.5±0.6 ^###^	**< 0.0001**

BMI: Body mass index; LVEF: Left ventricular ejection fraction. Bonferroni post-hoc test: * p ≤ 0.05 *vs* CTRL; ^#^ p ≤ 0.05, ^##^ p ≤ 0.01, ^###^ p ≤ 0.001 *vs* AVSc.

## Data Availability

All *in vitro* data supporting the findings of this study are available within the paper and its Supplementary Information. The anonymized patient-level data are available under restricted access in accordance with the institutional ethics approval. Access can be obtained for research related to cardiovascular diseases by not-for-profit organizations, providing a local institutional review board approval and a letter of collaboration with the study investigators. Requests can be made to the corresponding author.

## Abbreviations

ACTA2: actin alpha 2
AdDMEM: advanced Dulbecco's modified Eagle's medium
AS: aortic valve stenosis
AVR: aortic valve replacement
AVSc: aortic valve sclerosis
BCA: bicinchoninic acid
BgAA: 1β-glycerophosphate and ascorbic acid
CD31: platelet endothelial cell adhesion molecule
COL1A1: collagen 1 alpha 1
CTRL: control
EACTS: European Association for Cardio-Thoracic Surgery
EndMT: endothelial-to-mesenchymal transition
ESC: European Society of Cardiology
FCAVD: fibro-calcific aortic valve degeneration
FLT3-ligand: Fms-related Tyrosine Kinase 3 ligand
GROα: Growth Regulated Oncogene-α
GROβ: Growth Regulated Oncogene-β
Htert: human telomerase reverse transcriptase
IFNα: Interferon- α
IFNβ: Interferon-β
INFγ: Interferon-γ
IL-1α: Interleukin-1α
IL-1β: Interleukin-1β
IL-10: Interleukin-10
IL-13: Interleukin-10
LVEF: left ventricular ejection fraction
M200: medium 200
MCP-1: Monocyte Chemoattractant Protein-1
MIP-1β: Macrophage Inflammatory Protein-1β
NM: normal media
PDGF-AB/BB: Platelet-derived Growth Factor AB/BB
PD-L1/B7-H1: Programmed cell death 1 ligand 1
POM: pro-osteogenic media
ProCol1a1: Pro-collagen 1a1
RANTES: Regulated on Activation, Normal T cell Expressed and Secreted
SDS: sodium dodecyl sulfate
SNAI1: Snail Family Transcriptional Repressor 1
SNAI2: Snail Family Transcriptional Repressor 2
TAVI: transcatheter aortic valve implantation
TGFβ1: Transforming Growth Factor beta 1
TGFβ2: Transforming Growth Factor beta 2
TNFα: Tumor Necrosis Factor-α
TRAIL: Tumor Necrosis Factor-Related Apoptosis-Inducing Ligand
VECs: valve endothelial cells
VICs: valve interstitial cells
