# Supplementary material for: Circulating cytokines as triggers of endothelial dysfunction and sex-specific interstitial cell response in fibrocalcific aortic valve disease
Source: Int J Biol Sci. 2026 Apr 23;22(9):4847–62. doi: 10.7150/ijbs.125469 (PMC13182535; doi:10.7150/ijbs.125469)
Supplement: Supplementary file 1 — Supplementary figures and tables. [file ijbsv22p4847s1.pdf]

Circulating cytokines as triggers of endothelial dysfunction and sex-specific interstitial cell response in fibrocalcific aortic valve disease

Vincenza Valerio[1*], Francesca Bertolini[1*], Valentina Rusconi[1,2], Mattia Chiesa[1], Ilaria Massaiu[1], Paola Gripari[1], Valentina Mantegazza[1], Cristina Gatto[3,4], Michele Ciccarelli[3], Arianna Galotta[1], Alice Bonomi[1], Marco Zanobini[1], Marco Agrifoglio[1,5] Veronika A. Myasoedova[1], and Paolo Poggio[1,5#]

[1] Centro Cardiologico Monzino IRCCS, 20138 Milan, Italy.

[2] University of Naples "Federico II", Department of Pharmacy, 80138 Naples, Italy.

[3] University of Salerno "Scuola Medica Salernitana", Department of Medicine, Surgery and Dentistry, 84081 Baronissi, Italy.

[4] University of Salerno "Scuola Medica Salernitana", Scuola di Specializzazione in Patologia Clinica e Biochimica Clinica, 84081 Baronissi, Italy.

[5] University of Milan, Department of Biomedical, Surgical, and Dental Sciences, 20122 Milan, Italy.

* These Authors contributed equally to the present work

# Correspondence

Paolo Poggio, PhD (ORCID: 0000-0002-7225-3379)

Associate Professor

Department of Biomedical, Surgical, and Dental Sciences, University of Milan

Via della Commenda 10, 20122 Milan, Italy

Email: paolo.poggio@unimi.it

Unit for the Study of Aortic, Valvular, and Coronary Pathologies

Centro Cardiologico Monzino IRCCS

Via privata Carlo Parea 4, 20138 Milan, Italy

TEL: +390258002853; Fax: +390258002342

Email: paolo.poggio@ccfm.it

Supplementary Material and Methods

iVICs Interferon-beta (IFNβ) stimulation

IFNβ was used to stimulate iVICs at concentrations ranging from 50 to 1000 U/mL. Plasma cytokine levels measured *in vivo* are in the picogram range and do not directly translate to effective concentrations in cell culture, as *in vitro* systems lack the dynamic processes of transport, binding, and rapid clearance present *in vivo*. To achieve receptor engagement and activate downstream signaling pathways, higher cytokine concentrations are typically required *in vitro*. Accordingly, concentrations in the range of hundreds to thousands of units per milliliter (U/mL) are commonly used in immunological cell culture studies to elicit measurable biological responses. In this study, 500 U/mL was selected as a baseline concentration based on previous evidence demonstrating effective activation of the JAK/STAT in cardiac fibroblasts [1], a pathway implicated in VIC calcification [2]. In consequence, lower (50 U/mL) and higher (1000 U/mL) concentrations were also tested to explore cellular responses below and above the baseline stimulation levels.

References

1. Bolivar S, Anfossi R, Humeres C, Vivar R, Boza P, Munoz C, et al. IFN-beta Plays Both Pro- and Anti-inflammatory Roles in the Rat Cardiac Fibroblast Through Differential STAT Protein Activation. Front Pharmacol. 2018; 9: 1368.

2. Parra-Izquierdo I, Sanchez-Bayuela T, Castanos-Mollor I, Lopez J, Gomez C, San Roman JA, et al. Clinically used JAK inhibitor blunts dsRNA-induced inflammation and calcification in aortic valve interstitial cells. FEBS J. 2021; 288: 6528-42.

Supplementary Tables

Table S1. Primers Sequences. Sequences of forward and reverse primers used to amplify the gene targets.

Gene name	Gene Symbol	Forword primer sequence	Reverse primer sequence
**Actin α2**	*ACTA2*	AGA GTT ACG AGT TGC CTG ATG	CTG TTG TAG GTG GTT TCA TGG A
**Collagen 1 A 1**	*COL1A1*	GGA CAC AGA GGT TTC AGT GG	CCA GTA GCA CCA TCA TTT CC
**Interleukin 1β**	*IL 1B*	CAG CCA ATC TTC ATT GCT CAA G	GAA CAA GTC ATC CTC ATT GCC
**Interleukin 6**	*IL6*	TTC TGT GCC TGC AGC TTC	GCA GAT GAG TAC AAA AGT CCT GA
**Ribosomal Protein L32**	*RPL32*	AAC GTC AAG GAG CTG GAA	GGG TTG GTG ACT CTG ATG G
**Runt-Related transcription factor 2**	*RUNX2*	CTT CAC AAA TCC TCC CCA AGT	AGG CGG TCA GAG AAC AAA C
**Snail Family Transcriptional Repressor 1**	*SNAI 1*	GGC TGC TAC AAG GCC AT	GCA CTG GTA CTT CTT GAC ATC T
**Snail Family Transcriptional Repressor 2**	*SNAI 2*	AGG ACA CAT TAG AAC TCA CAC G	CAG ATG AGC CCT CAG ATT TGA C
**Transforming Growth Factor β 1**	*TGFB1*	GTT CAG GTA CCG CTT CTC G	CCG ACT ACT ACG CCA AGG A
**Transforming Growth Factor β 2**	*TGFB2*	TCA ATG TAA AGT GGA CGT AGG C	TGA GTC ACA ACA GAC CAA CC

Table S2. Results from differential expressed gene analysis between controls, aortic valve sclerosis (AVSc) subjects, and severe aortic stenosis (AS) patients. List of the differentially expressed genes with the obtained values of log fold change (logFC) and pValue (Green highlighted pValue < 0.05) between AVSc *vs* CTRL subjects and AS *vs* AVSc patients.

	AVSc vs. CTRL		AS vs. AVSc		AS vs. CTRL	
**Protein**	**logFC**	**pValue**	**logFC**	**pValue**	**logFC**	**P.Value**
CD40	0.08	0.616	-0.31	0.054	-0.23	0.147
EGF	-0.14	0.320	-0.16	0.271	-0.31	0.036
Eotaxin	0.04	0.791	-0.37	0.020	-0.32	0.036
FGF_basic	0.05	0.741	-0.12	0.464	-0.06	0.682
Flt_3_Lig	0.16	0.236	-0.56	0.000	-0.40	0.004
G_CSF	0.04	0.753	-0.26	0.051	-0.22	0.098
GM_CSF	0.03	0.870	-0.10	0.543	-0.07	0.652
Granzyme_B	0.10	0.515	-0.28	0.077	-0.18	0.256
GRO_alpha	0.47	0.003	-0.27	0.081	0.19	0.220
GRO_beta	0.03	0.834	-0.19	0.212	-0.16	0.294
IFN_alpha	0.12	0.163	-0.21	0.012	-0.10	0.245
IFN_beta	0.09	0.557	-0.01	0.924	0.08	0.622
IFN_gamma	0.10	0.507	-0.08	0.592	0.02	0.900
IL_1_alpha	0.19	0.209	-0.40	0.011	-0.20	0.190
IL_1_beta	0.30	0.046	-0.46	0.002	-0.16	0.282
IL_1_RA	0.18	0.246	0.01	0.957	0.19	0.224
IL_10	-0.01	0.972	0.09	0.557	0.09	0.578
IL_12	0.02	0.885	-0.18	0.144	-0.16	0.184
IL_13	-0.11	0.465	0.11	0.464	0.00	0.995
IL_15	-0.08	0.576	-0.18	0.188	-0.26	0.060
IL_17_A	-0.02	0.764	-0.08	0.211	-0.10	0.118
IL_17_E	-0.24	0.128	-0.02	0.924	-0.26	0.105
IL_3	0.12	0.425	0.12	0.392	0.24	0.097
IL_33	0.24	0.115	-0.26	0.094	-0.02	0.908
IL_4	0.09	0.346	-0.08	0.395	0.01	0.930
IL_5	0.17	0.278	-0.07	0.669	0.10	0.511
IL_6	0.22	0.149	-0.23	0.137	-0.01	0.955
IL_7	0.04	0.808	-0.16	0.277	-0.13	0.393
IL_8	0.13	0.390	-0.17	0.271	-0.04	0.802
IL2_r2	0.07	0.631	-0.24	0.102	-0.17	0.240
IP_10	0.03	0.863	0.12	0.455	0.14	0.355
MCP_1	-0.11	0.481	-0.52	0.001	-0.62	0.000
MIP_1_alpha	0.00	0.982	0.06	0.711	0.06	0.726
MIP_1_beta	0.10	0.521	-0.34	0.034	-0.24	0.132
MIP_3_alpha	0.16	0.296	-0.29	0.060	-0.13	0.393
MIP_3_beta	0.22	0.162	0.03	0.840	0.25	0.109
PD_L1_B7_H1	0.03	0.865	-0.09	0.555	-0.07	0.671
PDGF_AA_r2	0.02	0.914	-0.18	0.240	-0.17	0.281
PDGF_AB.BB	-0.08	0.586	-0.19	0.225	-0.27	0.077
RANTES	0.15	0.329	-0.28	0.070	-0.13	0.394
TGF_alpha	-0.03	0.825	0.14	0.351	0.11	0.471
TNF_alpha	0.10	0.443	0.19	0.161	0.30	0.030
TRAIL	0.03	0.827	-0.35	0.029	-0.31	0.046
VEGF	-0.02	0.905	0.07	0.668	0.05	0.755

Table S3. Statistical summary of two-way ANOVA results for Figure 2. Statistical parameters for the experimental data presented in Figure 2 (Panels 2C-J) specifically examining the effects of Treatment, Time, and their Interaction. It provides the Sum of Squares (SS), effect size (partial eta squared, η^2_p), and pValues for each factor.

		Sum of squares (SS)	Partial eta squared (η^2p)	2way ANOVA pValue
**Panel 2C**	Interaction	0.0012	0.2371	0.1084
	Treatment	0.0096	0.7157	0.0005
	Time	0.0211	0.8461	<0.0001
**Panel 2D**	Interaction	0.0158	0.1972	0.1481
	Treatment	0.0578	0.4732	0.0134
	Time	0.3533	0.8459	<0.0001
**Panel 2E**	Interaction	0.006834	0.003790698	0.8492
	Treatment	0.006834	0.003790698	0.8492
	Time	830.8	0.997842891	<0.0001
**Panel 2F**	Interaction	1.199	0.436953353	0.0193
	Treatment	1.186	0.43427316	0.0198
	Time	231.7	0.993376064	<0.0001
**Panel 2G**	Interaction	0.004401	0.008131914	0.7805
	Treatment	0.004134	0.007642337	0.787
	Time	0.106	0.164903547	0.2193
**Panel 2H**	Interaction	0.04208	0.14501344	0.222
	Treatment	0.04293	0.147510566	0.2177
	Time	10.54	0.977002438	<0.0001
**Panel 2I**	Interaction	0.03413	0.046045087	0.503
	Treatment	0.03338	0.045078868	0.5077
	Time	19.38	0.964798303	<0.0001
**Panel 2J**	Interaction	0.7544	0.911904071	<0.0001
	Treatment	0.7508	0.911519037	<0.0001
	Time	20.19	0.996403275	<0.0001

Table S4. Results from differential expressed gene analysis between men and women with control aortic valve, aortic valve sclerosis (AVSc), and severe aortic stenosis (AS). List of the differentially expressed genes with the obtained values of log fold change (logFC) and pValue (Green highlighted pValue < 0.05) between men and women in the three categories.

	CTRL (M vs. F)		AVSc (M vs. F)		AS (M vs. F)	
**Protein**	**logFC**	**pValue**	**logFC**	**pValue**	**logFC**	**pValue**
TNF_alpha	0.32	0.088	0.44	0.015	0.48	0.017
RANTES	-0.09	0.663	-0.61	0.003	-0.55	0.024
IFN_beta	0.19	0.320	0.19	0.448	0.45	0.032
MIP_1_alpha	0.39	0.071	0.35	0.143	0.47	0.036
Granzyme_B	0.28	0.198	0.35	0.121	0.39	0.085
GM_CSF	0.07	0.771	0.04	0.829	-0.33	0.159
IL_7	0.25	0.271	0.28	0.151	-0.28	0.186
GRO_beta	0.16	0.521	-0.48	0.017	-0.25	0.224
IL_1_beta	0.61	0.002	0.41	0.046	0.26	0.241
IL_12	0.23	0.205	0.04	0.788	-0.22	0.261
Flt_3_Lig	-0.26	0.126	-0.16	0.444	0.22	0.303
IL_5	0.00	1.000	0.30	0.383	-0.19	0.304
MCP_1	0.18	0.489	-0.21	0.219	0.20	0.310
IL_13	0.37	0.081	0.44	0.042	0.19	0.388
IL_1_RA	0.26	0.161	0.12	0.631	-0.17	0.425
IL_17_A	0.04	0.754	0.03	0.692	-0.05	0.449
IL_33	0.37	0.062	0.03	0.896	-0.17	0.488
IL_6	0.25	0.198	0.41	0.101	0.14	0.502
Eotaxin	0.04	0.859	-0.16	0.469	-0.11	0.579
G_CSF	0.27	0.107	0.12	0.462	-0.13	0.585
MIP_1_beta	0.28	0.270	0.41	0.055	0.09	0.620
IFN_gamma	0.26	0.217	0.55	0.006	0.11	0.625
MIP_3_beta	0.04	0.861	0.27	0.255	-0.10	0.661
EGF	0.14	0.465	0.06	0.790	-0.09	0.670
IL_4	0.12	0.396	0.00	0.998	-0.05	0.685
PDGF_AA_r2	-0.25	0.220	-0.07	0.726	0.11	0.685
PD_L1_B7_H1	0.27	0.161	0.54	0.020	-0.10	0.687
TGF_alpha	0.31	0.145	0.44	0.092	-0.07	0.708
IL_1_alpha	0.30	0.183	0.50	0.021	0.07	0.734
MIP_3_alpha	0.30	0.179	0.58	0.010	0.08	0.740
CD40	0.02	0.891	0.17	0.224	-0.11	0.747
TRAIL	0.17	0.391	-0.12	0.393	0.10	0.752
VEGF	0.18	0.384	0.23	0.371	-0.07	0.763
IL_10	0.28	0.224	0.53	0.017	0.06	0.770
IL_8	0.00	0.998	0.07	0.784	-0.06	0.790
IP_10	0.23	0.306	0.19	0.427	-0.05	0.798
PDGF_AB.BB	0.43	0.037	0.13	0.426	0.06	0.836
IL_15	0.29	0.144	-0.12	0.545	-0.04	0.841
IL2_r2	0.12	0.582	0.11	0.564	-0.04	0.846
GRO_alpha	0.30	0.328	-0.33	0.041	-0.03	0.888
IFN_alpha	0.13	0.165	0.18	0.240	-0.01	0.921
IL_3	-0.26	0.158	-0.04	0.862	0.02	0.938
FGF_basic	0.07	0.758	-0.15	0.498	0.01	0.956
IL_17_E	0.43	0.191	0.23	0.227	0.00	0.985

Table S5. Statistical summary of two-way ANOVA results for Figure 4. Statistical parameters for the experimental data presented in Figure 4 (Panels 4I-L) specifically examining the effects of Treatment, Sex, and their Interaction. It provides the Sum of Squares (SS), effect size (partial eta squared, η^2_p), and pValues for each factor.

	Sum of squares (SS)		Partial eta squared (η^2p)	2way ANOVA pValue
**Panel 4I**	Interaction	0.2210	0.0978	0.3572
	Treatment	1.0760	0.3455	0.0206
	Sex	0.6171	0.2324	0.1286
**Panel 4J**	Interaction	2.0670	0.1473	0.2033
	Treatment	4.3110	0.2648	0.0500
	Sex	8.9100	0.4267	0.0165
**Panel 4K**	Interaction	0.1864	0.0836	0.4178
	Treatment	1.8980	0.4815	0.0056
	Sex	11.4800	0.8489	<0.0001
**Panel 4L**	Interaction	0.7944	0.1947	0.1147
	Treatment	0.7742	0.1907	0.1325
	Sex	4.3540	0.5700	0.0012

Table S6. Statistical summary of two-way ANOVA results for Figure 5. Statistical parameters for the experimental data presented in Figure 5 (Panels 5I-L) specifically examining the effects of Treatment, Sex, and their Interaction. It provides the Sum of Squares (SS), effect size (partial eta squared, η^2_p), and pValues for each factor.

		Sum of squares (SS)	Partial eta squared ($\eta^2 p$)	2way ANOVA pValue
**Panel 5I**	Interaction	3.8020	0.2940	0.0308
	Treatment	10.3500	0.5313	0.0011
	Sex	7.2820	0.4437	0.1648
**Panel 5J**	Interaction	0.1149	0.2811	0.0369
	Treatment	0.7983	0.7309	<0.0001
	Sex	2.8490	0.9065	<0.0001
**Panel 5K**	Interaction	0.6426	0.0775	0.4465
	Treatment	3.3210	0.3026	0.0278
	Sex	7.3460	0.4898	0.3983
**Panel 5L**	Interaction	0.0859	0.5935	0.0001
	Treatment	0.4843	0.8917	<0.0001
	Sex	0.4288	0.8794	0.0069

Table S7. Statistical summary of two-way ANOVA results for Figure 6. Statistical parameters for the experimental data presented in Figure 6 (Panels 4K-N) specifically examining the effects of Treatment, Sex, and their Interaction. It provides the Sum of Squares (SS), effect size (partial eta squared, $\eta^2{}_p$), and pValues for each factor.

		Sum of squares (SS)	Partial eta squared (η^2p)	2way ANOVA pValue
**Panel 6K**	Interaction	1.2530	0.2185	0.0849
	Treatment	4.7870	0.5165	0.0013
	Sex	6.5460	0.5936	0.0001
**Panel 6L**	Interaction	0.0500	0.1398	0.2219
	Treatment	0.0156	0.0481	0.5614
	Sex	0.4579	0.5981	0.0219
**Panel 6M**	Interaction	0.7690	0.2595	0.0495
	Treatment	3.7910	0.6334	0.0002
	Sex	3.8090	0.6345	0.0047
**Panel 6N**	Interaction	0.0654	0.0954	0.3671
	Treatment	0.1016	0.1407	0.2243
	Sex	0.2114	0.2541	0.0457

Supplementary Figures

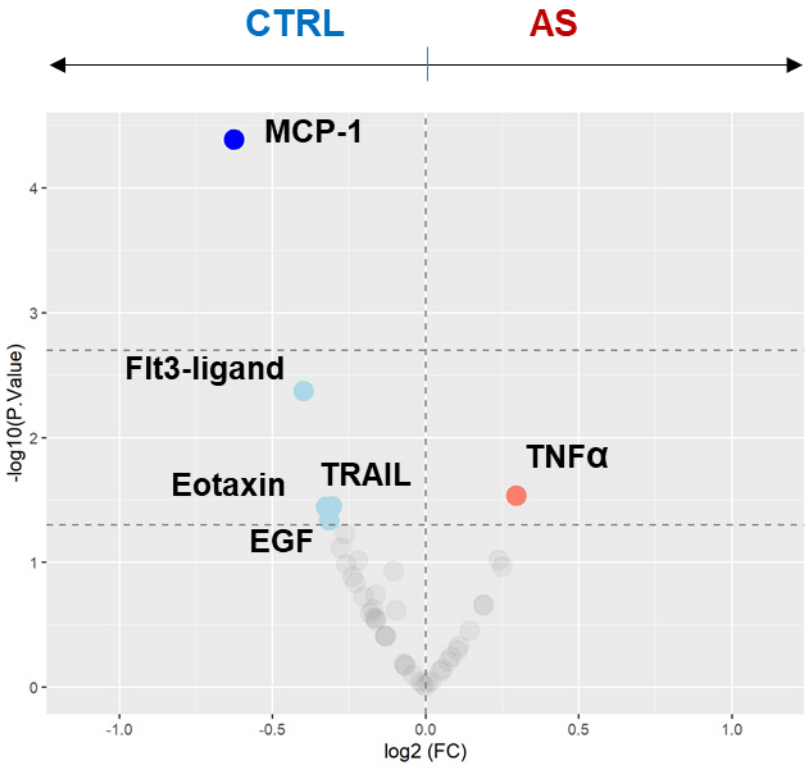

Figure S1. Serum cytokine profiling in stenotic (AS) *vs* control (CTRL) subjects. Volcano plot showing the differential levels of circulating cytokines in severe AS patients compared to CTRL. Colored dots indicate differentially expressed cytokines. Light color indicates cytokines with a pValue < 0.05 (dashed line at −log10 of 1.3), while dark color indicates a pValue < 0.002 (dashed line at −log10 of 2.7).

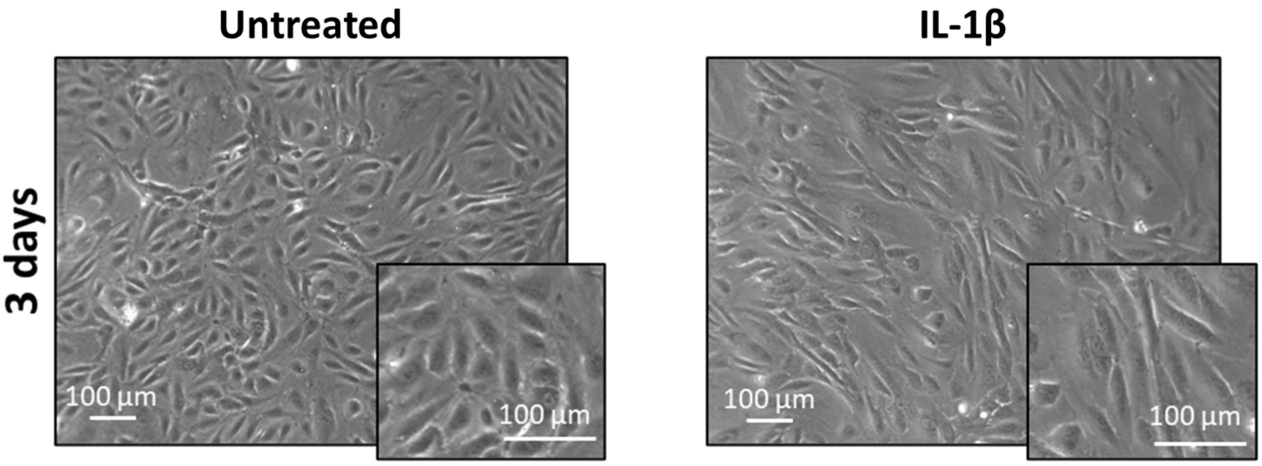

Figure S2. IL-1β-treated valve endothelial cells. Representative brightfield images showing valve endothelial cells (VEC), after 3 days in culture, in the absence of treatment (left panel) and after treatment with IL-1β (right panel). Magnification 20x; Scale bar: 100 μM.

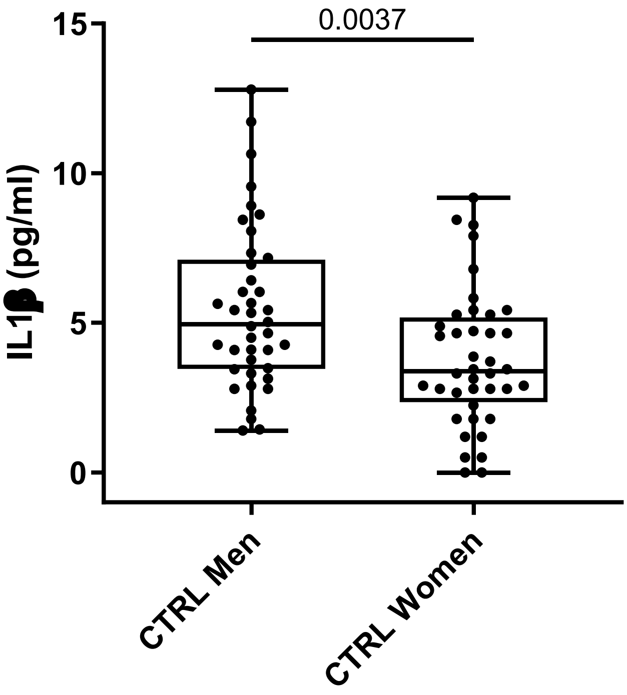

Figure S3. Circulating IL-1β levels in control men and women. Box and whisker plots showing circulating IL-1β serum levels in control men and women (CTRL; n = 40 for each group). The dashed line indicates the upper value of the IL-1β normal range (12 pg/mL). Data are presented as median ± interquartile range with minimum and maximum values. The represented pValue is an unpaired t-test.

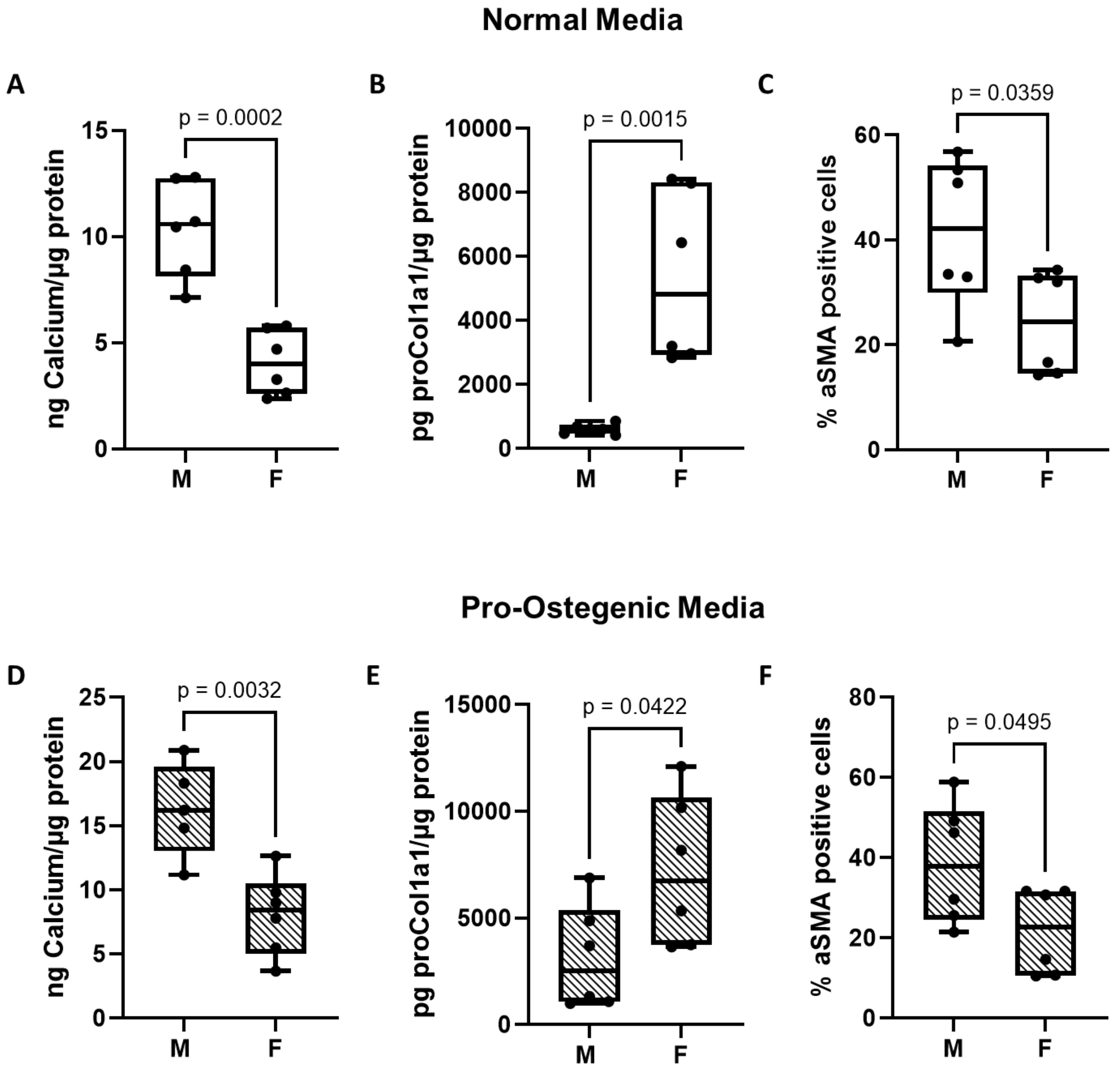

Figure S4. Sex-dependent differences in calcification potential, pro-collagen production and αSMA positivity of iVICs. Box and whiskers representing men's and women's calcium deposition, pro-collagen production and αSMA positivity in normal media (**A-B-C**) and (**D-E-F**) pro-osteogenic media (n = 6 for each group). Data are presented as median ± interquartile range with minimum and maximum values. All represented pValues are unpaired t-tests.

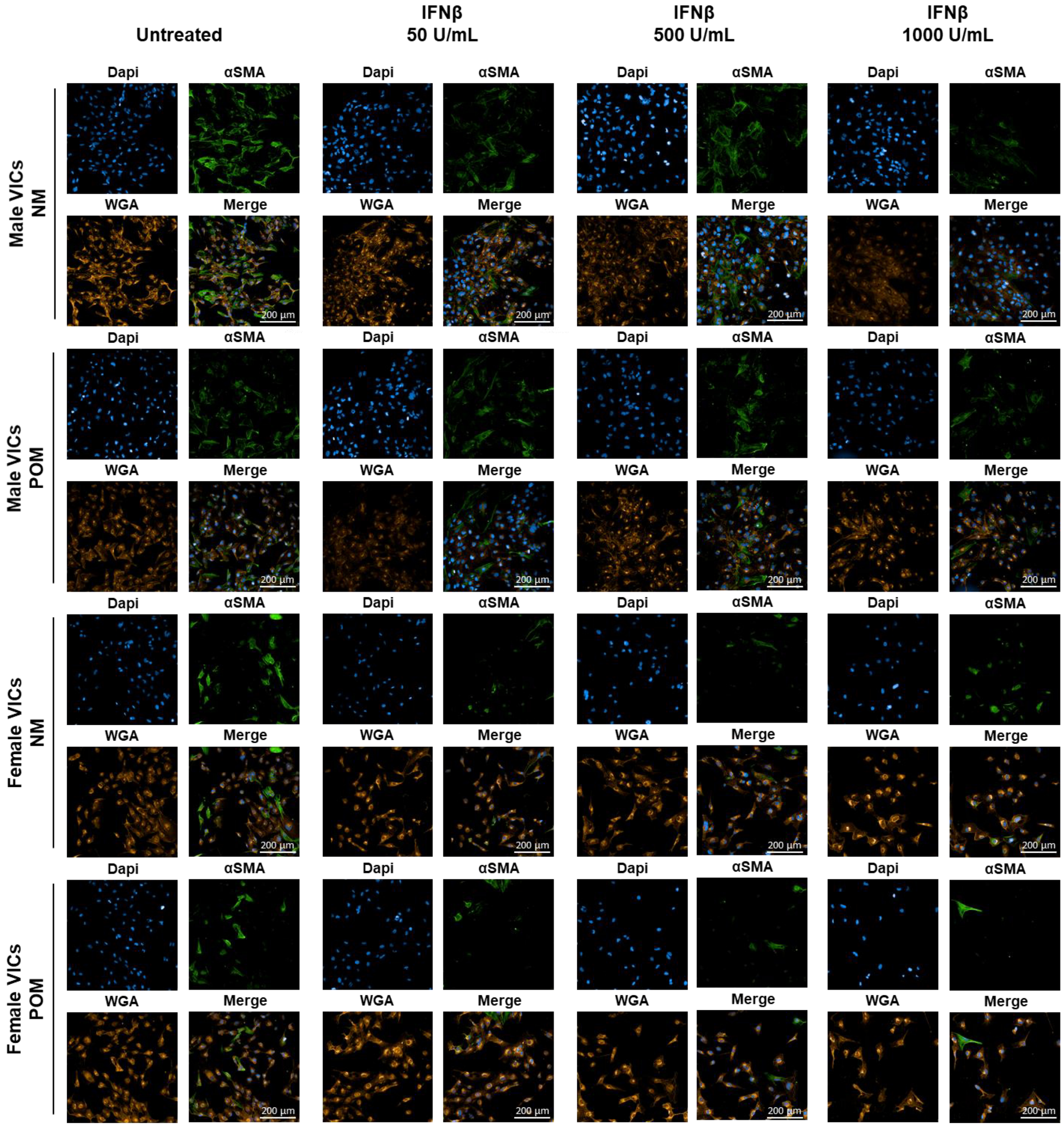

Figure S5. Smooth muscle actin (αSMA) expression and modulation by IFNβ in male and female iVICs. Representative immunofluorescence images showing αSMA (green) in male and female iVICs cultured in normal media (NM) and pro-osteogenic media (POM) supplemented with different concentrations of INFβ (0, 50, 500, and 1000 U/mL). Membranes were visualized with wheat germ agglutinin (WGA; orange) and nuclei with 4',6-diamidino-2-fenilindolo (DAPI; blue). Magnification 20x. Scale bar: 200 μM.

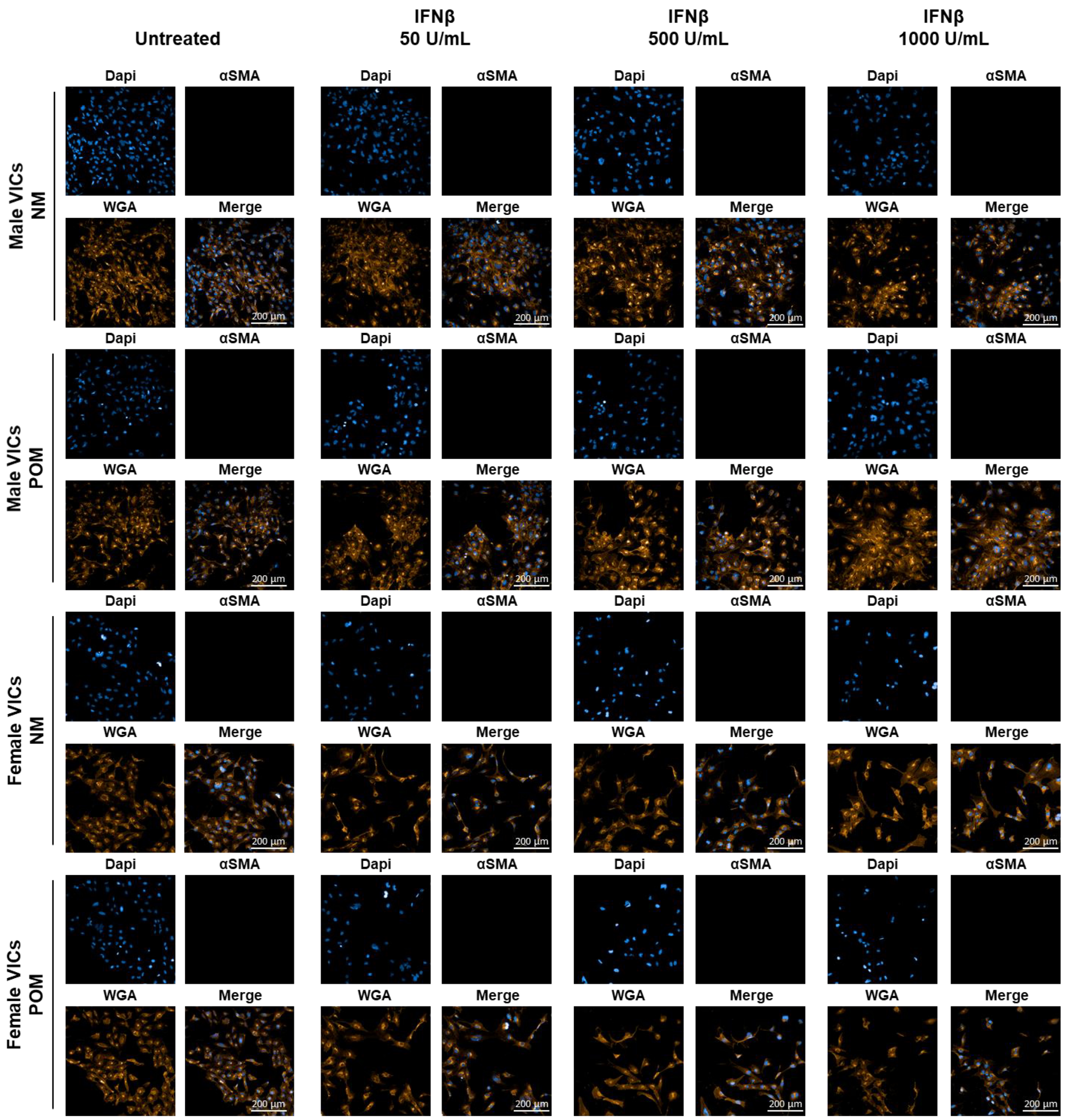

Figure S6. Immunofluorescence negative control. Representative immunofluorescence images using only secondary antibody (negative control; green). Membranes are visualized with wheat germ agglutinin (WGA; orange) and nuclei with 4',6-diamidino-2-fenilindolo (DAPI; blue). Magnification 20x. Scale bar: 200 μM.
